# Role of environmental pollutants-induced ferroptosis in pulmonary diseases

**DOI:** 10.3389/fmed.2025.1542275

**Published:** 2025-02-24

**Authors:** Long Yang, Yongkang Qiao, Zeyu Huang, Yuzhu Chen, Enxi Zhang, Zhiwei Liu, Yuxuan Wang, Shaobo Chen, Jinrui Dong, Bin Liu

**Affiliations:** ^1^Integrated Medical Center, Tianjin University, Tianjin, China; ^2^Key Laboratory of Cell Proliferation and Regulation Biology, Ministry of Education, Beijing Normal University, Zhuhai, Guangdong, China; ^3^Academy of Medical Engineering and Translational Medicine, School of Medicine, Tianjin University, Tianjin, China; ^4^School of Pharmaceutical Science and Technology, Tianjin University, Tianjin, China; ^5^Institute of Plateau and Cold-Resistant Environment and Cardiovascular Disease Prevention and Treatment, Specialty Medical Center of Chinese People’s Armed Police Force, Tianjin, China; ^6^Department of Infectious Diseases, Specialty Medical Center of Chinese People’s Armed Police Force, Tianjin, China

**Keywords:** ferroptosis, lung, environmental pollutants, iron, redox

## Abstract

Respiratory diseases rank among the foremost causes of mortality and disability globally, with long-term exposure to environmental pollutants playing a critical role in their onset and progression. Despite this, the underlying mechanisms and effective targeted treatments for these disorders remain poorly understood, highlighting an urgent need for focused research. Cell death, a programmed cellular response to external harmful stimuli, including ferroptosis—a recently identified form of iron-dependent programmed cell death—emerges as a pivotal process. Characterized by intracellular iron accumulation and lipid peroxidation, ferroptosis appears intricately linked to lung injury induced by environmental pollutants. This review examines the role of ferroptosis in lung diseases triggered by environmental factors, aiming to shed light on its specific pathophysiological mechanisms and potential as a therapeutic target. By deepening our understanding of the interactions between environmental pollution, ferroptosis, and lung damage, we hope to inform strategies for effective intervention.

## 1 Introduction

The respiratory system serves as a primary interface between the body and the external environment. It handles inhaled gases, pollutants, and pathogens, so pollutants in the environment can directly cause inflammation and damage to the lungs (1). It has been proved that particles (mainly PM2.5), ozone, carbon monoxide, nitrogen oxides and heavy metals in the air can directly impact lung function and damage lung tissue (2). These pollutants exert their toxicological effects mainly by disrupting the redox balance in the lung (3). In addition, working with ferrous materials such as silica and asbestos significantly impairs pulmonary function (4). Furthermore, with aging, there is an impairment in lung function among elderly people, which amplifies the susceptibility to environmental harm and health risks such as air pollution, infections, and climate change (5). Consequently, it is imperative to urgently investigate the exact mechanisms underlying lung diseases.

Cell death is essential for the normal development of cells and organisms, as well as for the maintenance of homeostasis in the internal environment. Once cell death is dysregulated, it leads to a variety of pathological consequences (6). In recent years, researchers have proposed new types of cell death according to different morphological characteristics of cell death, such as necrotic apoptosis, autophagic death, scorch death and ferroptosis (7). Ferroptosis, a type of regulatory cell death discovered by Dixon et al., plays a pivotal role in tumors, neurodegenerative diseases, brain injury, ischemia-reperfusion injury, atherosclerosis, diabetes, inflammatory bowel disease, and acute renal failure (8, 9). In recent decades, the incidence of malignant and non-malignant respiratory diseases has increased dramatically due to environmental problems caused by anthropogenic and natural factors (10, 11). While apoptosis and necrosis are well-established mechanisms in the pathogenesis of various pulmonary diseases (12), ferroptosis—a form of regulated cell death driven by iron accumulation and lipid peroxidation—has emerged as a distinct contributor to the development and progression of conditions such as chronic obstructive pulmonary disease (COPD), pulmonary fibrosis, and lung cancer (13, 14). Unlike traditional cell death pathways, ferroptosis is characterized by the oxidative degradation of membrane lipids and iron dysregulation, processes closely linked to heightened oxidative stress in pulmonary pathophysiology (15). The discovery of ferroptosis may provide new insights into the pathophysiology of respiratory diseases.

The ability of environmental pollutants to induce various adverse consequences in human health has been widely recognized, including inflammation, apoptosis, necrosis, pyroptosis and autophagy (16, 17). The occurrence of ferroptosis is the result of the explosive accumulation of iron in cells and the peroxidation of phospholipids, which leads to oxidative stress and the accumulation of metabolites, and eventually leads to cell and tissue damage (8). Numerous studies have shown that fine particulate matter, heavy metals, and organic substances can trigger ferroptosis, which is closely related to lipid, iron, and amino acid metabolism (18). Given the growing evidence that ferroptosis is associated with severe diseases such as heart failure, chronic obstructive pulmonary disease, liver injury, Parkinson’s disease, Alzheimer’s disease, and cancer (19, 20). It is of great clinical and societal value to investigate the role of ferroptosis in pollution-induced lung diseases.

In this review, we summarize the main pathophysiological mechanisms of ferroptosis, the pathways and complex signaling molecules of ferroptosis in lung diseases dominated by environmental factors, including special environment and new environmental pollutants. Finally, we provide a perspective on future research directions and strategies to prevent pollution-induced ferroptosis. By enhancing our understanding of this novel form of cell death and developing effective preventive measures, we can mitigate the adverse effects of environmental contaminants and protect human and environmental health.

## 2 Mechanism of ferroptosis

**FIGURE 1:**
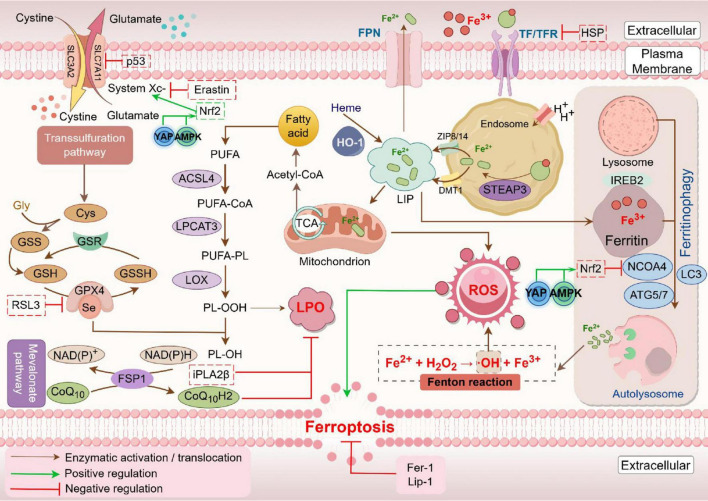
Mechanism of ferroptosis. Fe^2+^ levels and the Fenton reaction are crucial in driving reactive oxygen species (ROS) production and lipid oxidation in ferroptosis. In this process, iron-containing proteins produce superoxide (O_2_^⋅^), which can be further converted to hydrogen peroxide (H_2_O_2_). O_2⋅_ and H_2_O_2_ attack the iron-sulfur clusters and heme groups, resulting in the release of Fe^2+^. The released Fe^2+^ catalyzes the Fenton reaction to generate hydroxyl radicals (OH^⋅^), which react with lipid molecules (LH) to form lipid peroxyl radicals (LOO⋅). Additionally, lipoxygenases (LOXs) can oxidize LH to lipid hydroperoxides (LOOH), which can also be converted to LOO⋅ by Fenton reaction. Accumulation of these lipid peroxyl radicals eventually induces ferroptosis. This process is inhibited by iron chelators such as deferoxamine (DFO), as well as by glutathione peroxidase 4 (GPX4) (Created by Figdraw).

The pathogenesis and pathophysiological characteristics of ferroptosis are being studied with increasing attention since it was identified in 2012 as an iron-dependent kind of non-apoptotic cell death (20). Ferroptosis can occur through two major pathways: the extrinsic or transporter-dependent pathway, and the intrinsic or enzyme-regulated pathway (21). Ferroptosis is caused by a redox imbalance between oxidants and antioxidants, which is driven by the abnormal expression and activity of multiple redox-active enzymes that produce or detoxify free radicals and lipid oxidation products (22) (Figure 1).

### 2.1 Iron metabolism and ferroptosis

Iron is an essential trace element essential for hemoglobin, myoglobin, and various enzymatic functions, including oxygen transport, electron transfer in mitochondria, and DNA synthesis (23). Iron homeostasis relies on proteins such as transferrin (TF), ferroportin (FPN), ferritin, and regulatory factors like Hepcidin (13, 24). Fe^3+^ enters cells via TF and transferrin receptors, is reduced to Fe^2+^ by STEAP3, and stored in labile iron pool (LIP) by the zinc-iron regulatory protein family 8/14 (ZIP8/14) or transported by divalent metal transporter 1 (DMT1) (25). However, intracellular iron ions are mainly stored in ferritin (FT), only a small amount of Fe^2+^ is stored in LIP (26). FPN mediates iron efflux, critical for systemic iron regulation, while Hepcidin decreases iron export by binding FPN (7, 13, 27, 28).

Ferroptosis is an iron-dependent, oxidative cell death characterized by excessive Fe^2+^ accumulation, lipid peroxides (LPO), and reactive oxygen species (ROS) (29). Iron apoptosis differs markedly from apoptosis, necrosis and autophagy in its morphology, biochemistry and regulatory mechanisms. It manifests distinct morphological changes such as plasma membrane rupture and mitochondrial damage, differentiating it from apoptosis and necrosis (30, 31). Additionally, as the metabolic center of the organism, reduced or disappeared mitochondrial crista, ruptured mitochondrial outer membrane and concentrated mitochondria are noticed in ferroptosis cells (8). The main biochemical features include elevated lipid peroxidation, increased intracellular iron, glutathione depletion, and reduced glutathione peroxidase 4 (GPX4) activity (8, 32–34). Ferroptosis can be triggered by agents like erastin and cisplatin, while inhibited by iron chelators such as deferoxamine (DFO) (35, 36). In addition, regulatory factors, including P53, heat shock proteins (HSPs), nuclear factor erythroid-derived 2-like 2 (NRF2), heme ferroptosis through modulation of iron metabolism (37).

### 2.2 Redox homeostasis

Ferroptosis primarily results from disrupted redox homeostasis, leading to the generation of hydrogen peroxide, which reacts with excess iron to form hydroxyl radicals (⋅OH) (38). This cascade reaction initiates lipid peroxidation of polyunsaturated fatty acids (PUFAs) in cell membranes, ultimately causing membrane damage (39). Increased Fe^2+^ levels may arise from transferrin-mediated uptake or ferritin autophagy, a process regulated by nuclear receptor coactivator 4 (NCOA4), which facilitates the conversion of ferritin from Fe^3+^ to Fe^2+^ in the lysosomes, releasing free iron into the cytoplasm-a phenomenon known as ferritinophagy (40). It has been shown that NCOA4 knockout diminishes cellular sensitivity to ferroptosis (41), while iron-binding regulatory protein 2 (IREB2) serves as a key regulator of iron metabolism and its silencing further reduces ferroptosis susceptibility (8, 42–44).

The cellular antioxidant defense against ferroptosis involves the cystine-glutamate antiporter (System Xc-) and GPX4. System Xc-, composed of SLC7A11 and SLC3A2, facilitates the exchange of extracellular cystine for intracellular glutamate, with cystine subsequently reduced to cysteine for glutathione (GSH) synthesis (45). GSH functions as a critical reducing agent and serves as a cofactor for GPX4, which catalyzes the reduction of lipid peroxides to less harmful lipid alcohols. This process effectively prevents the accumulation of lipid peroxides, thereby protecting cellular membranes from oxidative damage (46). Elevated extracellular glutamate can inhibit System Xc-, promoting ferroptosis, particularly in cells deficient in SLC7A11 or GPX4, while their overexpression confers resistance (47). Inhibitors targeting System Xc- and GPX4 significantly enhance ferroptosis (48, 49). For instance, Erastin blocks GSH synthesis by impeding cystine uptake via SLC7A11, thereby inhibiting GPX4 activity. RAS-selective lethality protein 3 (RSL3) covalently binds and inhibits GPX4, leading to lipid peroxide accumulation. Additionally, ferroptosis suppressor protein 1 (FSP1) operates similarly to GPX4, collaborating with it to eliminate lipid peroxides, thereby offering cellular protection against ferroptosis (50). This multifaceted regulatory framework highlights the complex interplay between iron metabolism and oxidative stress in ferroptosis, underscoring potential therapeutic targets for mitigating iron-related cellular damage (51).

### 2.3 Peroxidation of polyunsaturated fatty acids in cell membrane

Lipid peroxidation, a key initiator of ferroptosis, involves the oxidation of PUFAs by ROS and lipoxygenase (LOX), producing malondialdehyde (MDA) and 4-hydroxynonenal (4-HNE) (52). This process disrupts membrane integrity by altering cell membrane structure, increasing permeability, and ultimately leading to rupture. Enzymes like Acyl-CoA synthetase long-chain family member 4 (ACSL4) and lysophosphatidylcholine acyltransferase 3 (LPCAT3) are critical to this pathway. ACSL4 activates free PUFAs to PUFA-CoA (53), which LPCAT3 esterifies to form PUFA-phospholipids (PUFA-PL) that integrate into cell membranes (15). These PUFA-PLs are highly susceptible to peroxidation via LOX, leading to phospholipid hydroperoxide accumulation, which, once above a threshold, triggers ferroptosis (54).

Phosphatidylethanolamine (PE) lipid peroxidation is particularly prominent in cellular ferroptosis, with ACSL4 facilitating PE esterification and LPCAT3 acylating PE lysophospholipids (39, 53). 15-LOX catalyzes oxidation at specific carbon sites (positions 15 and 17) in fatty acids, while the Ca^2+^-independent phospholipase A2 (iPLA2β) regulates ferroptosis by hydrolyzing peroxidized phospholipids, thus mitigating ferroptosis progressio (55, 56). Intriguingly, in the presence of iron, phospholipid hydroperoxides generate free lipid radicals, which can abstract protons from neighboring PUFAs, initiating a chain reaction of lipid peroxidation that may propagate ferroptosis between cells (54, 57, 58).

Furthermore, oxidoreductase myristoylated FSP1, recruited to the cell membrane, uses NAD(P)H to reduce coenzyme Q10 (CoQ10) to ubiquinone, capturing lipid peroxidation radicals and interrupting lipid peroxidation cascades (51). Similarly, mitochondrial dihydroorotate dehydrogenase (DHODH) reduces CoQ10, working alongside GPX4 to neutralize mitochondrial lipid peroxides, thereby suppressing ferroptosis (59). Ferroptosis inhibitors like Ferrostatin-1 (Fer-1) and Liproxstatin-1 (Lip-1) act as potent antioxidants that sequester lipid peroxidation radicals, offering protective effects against ferroptosis by inhibiting lipid oxidative damage (60, 61).

## 3 Environmental pollution-induced ferroptosis in lung diseases

Environmental pollution arises when humans release substances or energy into the environment beyond its self-purification capacity, degrading environmental quality and threatening human health, ecosystems, and property (62). Air pollution, characterized by high concentrations of pollutants like particulate matter (PM), ozone, volatile organic compounds, carbon monoxide (CO), and nitrogen oxides (NOx), which have serious respiratory health effects and are estimated to cause 90,000 deaths globally each year (63). Epidemiological studies have demonstrated that a significant number of patients are admitted to hospitals annually due to a decline in air quality (64, 65). Fine particulate matter (PM), particularly PM2.5 (66, 67), the primary component of air pollution (68), can penetrate deeply into the lungs, reaching alveolar ducts and causing structural and functional lung tissue damage (69). This can weaken immune function, disrupt the autonomic nervous system, and significantly elevate mortality rates, particularly among individuals with chronic illnesses (70, 71). Mechanisms of lung injury linked to PM2.5 include its role as a metal carrier, production of reactive oxygen species (ROS), damage to the airway mucosa, and alterations in the lung microbiome (72, 73).

Multiple studies have proved the role of environmental pollution-induced ferroptosis in lung diseases (Table 1). Environmental pollution, particularly PM2.5, is strongly linked to ferroptosis in lung injury. Studies identify ferroptosis as a key risk factor in PM2.5-induced lung damage, with notable cellular changes such as mitochondrial contraction, membrane thickening, and wedge-pattern ruptures. PM2.5 exposure triggers increased TFRC expression, iron transport, and accumulation, leading to elevated ROS levels and GPX4 activation, culminating in ferroptosis (70). Additionally, PM2.5 activates the AMPK-Beclin1 autophagy pathway, reducing cell viability, increasing lipid peroxidation, disrupting iron metabolism proteins, and downregulating SLC7A11 in a dose-dependent fashion (74). Ferroptosis inhibitors (e.g., DFO, Fer-1) partially restore cellular health by reducing lipid peroxidation and iron levels. Furthermore, PM2.5-induced ferroptosis is mediated through the PI3K/Akt/NRF2 pathway, with agents like sipramine showing protective effects (75). These findings underscore the therapeutic potential of targeting ferroptosis to treat PM2.5-related lung damage.

**TABLE 1:** Environmental pollution-induced ferroptosis in lung diseases.

Pollutants	Models of diseases	Mechanism	References
PM2.5	Lung injury	NRF2/SLC7A11/GPX4 axis	(70)
		PI3K/Akt/NRF2 axis	(75)
Dust mite	Mouse asthma model	Dysmorphic small mitochondria	(77)
PM2.5	Exacerbation of asthma	Activation of the NF-κB signaling pathway, increased iron content	(74, 85)
PM2.5	Acute exacerbation of COPD	Disruption of REDOX homeostasis	(94)
PM/PM2.5	Acute lung injury (ALI)	NRF-2 mediated ferroptosis	(96, 99)
PM2.5	Pneumonia	USP3-SIRT3-P53 mediated ferroptosis	(98)
BeSO_4_	ALI	Lipid peroxidation	(106)
Nanoparticles and cadmium	ALI	ATG5-dependent ferroptosis	(107)
PM2.5	Exacerbation of pulmonary fibrosis	HO-1-mediated oxidative stress, NCOA4-mediated ferritin autophagy	(114)
SiO_2_	Silicosis	TGF-β mediated macrophages ferroptosis	(116, 120)
Radiation	pulmonary fibrosis	NRF-2 and TGF-β mediated ferroptosis	(123)
Bituminous coal combustion	Lung cancer	TXN2 mediated ferroptosis	(133)

### 3.1 Environmental pollution-induced ferroptosis in inflammatory lung diseases

Bronchial asthma is a reversible, chronic inflammatory disease of the airways. Currently, there is a global population of 300 million individuals who are afflicted with asthma, and there has been a notable rise in its occurrence among young individuals (76). In house dust mite-induced mouse asthma model, pulmonary lipid peroxidation and ROS levels are increased, suggesting that ferroptosis may be involved in the pathogenesis of asthma (77). It was found that BEAS-2b cells treated with LPS and interleukin (IL)-13 induced increased expression of inflammatory factors such as IL-6 and IL-33, consistent with the pathological changes in asthma. The cells exhibited fragmentation of mitochondrial cristae, swelling, and increased vacuolation under the microscope, as well as elevated ROS levels, which are hallmarks of ferroptosis. Ferroptosis inhibitor Lip-1 can significantly reduce the LPS or IL-13-induced cell damage, and alleviate the down-regulation of GPX4 and SLC7A11 (78, 79). Another study found that human airway epithelial cells stimulated with IL-13 showed increased expression of 15-LOX and formed a PEBP1/15-LOX complex with phosphatidylethanolamine-binding protein 1 (PEBP1). PEBP1 further catalyzed the oxidation of PE-AA, leading to ferroptosis. The co-localization between PEBP1 and 15-LOX in airway epithelial cells is enhanced in individuals with asthma compared to healthy controls, and there is a substantial correlation with the expression of airway inflammation markers (55). In addition, in ovalbumin (OVA)-induced mouse asthma model, the expression of IL-13 aggravated the oxidation of PUFA-PE by activating the expression of 15-LOX and 5-LOX, thereby inducing ferroptosis (79, 80). Ferroptosis inhibitor Fer-1 or antioxidant N-Acetyl-L-cysteine could significantly ameliorate IL-6-induced lipid peroxidation and iron homeostasis in human bronchial epithelial cells.

Additionally, asthma is a heterogeneous genetic disease, influenced by both genetic and environmental conditions (especially PM2.5 exposure) (81). Numerous epidemiologic investigations have shown that PM2.5 exposure is closely associated with the progression of several respiratory diseases, leading to airway inflammation, decreased lung function, and the exacerbation of asthma (82–84). Pre-clinical studies have shown that PM2.5 aggravates the oxidative stress and inflammatory response in experimental mice asthma model by activating the nuclear factor κB (NF-κB) signaling pathway (85), indicating the role of PM2.5 in the pathophysiology of asthma. Studies have found that PM2.5 exposure can lead to increased iron content, lipid peroxidation and REDOX imbalance in endothelial cells, leading to ferroptosis and the secretion of inflammatory factors (74). However, Fer-1 and iron chelator DFO can reduce endothelial cell death and inflammatory factor secretion (86). Therefore, the inhibition of lipid peroxidation and ferroptosis in airway epithelial cells and inflammatory cells such as eosinophils may provide new insights into the development of drugs treating environmental pollution-exacerbated asthmatic patients with strong specificity and less adverse reactions.

COPD is a chronic lung disease characterized by progressive and irreversible airflow limitation. The substantial morbidity and death associated with COPD imposes a considerable economic burden (87). COPD is presently the third leading cause of death globally, responsible for 8.2% of all fatalities due to diseases (62). Although there are other factors that contribute to COPD, smoking continues to be a predominant risk factor for the development of COPD (88). Whole cigarette smoke condensate (WCSC) treatment induce mitochondrial abnormalities, such as condensation or swelling, increased membrane density, reduced or absent crista, as well as rupture of the outer membrane, showing the characteristics of ferroptosis in human bronchial epithelial BEAS-2b cells (89). Enrichment analysis of differential expressed genes showed that WCSC could activate ferroptosis-related signal pathway (90). Exposure to cigarette smoke (CS) caused lipid peroxidation in human bronchial epithelial cells (HBE) over a 24-h period. The cells displayed morphological changes associated with ferrocytic apoptosis under electron microscopy. The iron chelator DFO, along with ferroptosis inhibitors Fer-1 and Lip-1, effectively reduced CS-induced lipid peroxidation, Fe^2+^ elevation, and cell death. Nevertheless, both necrosis inhibitor necrostatin-1 and apoptosis inhibitor zVAD-FMK failed to improve the cell death caused by CS (91). *In vivo* experiment confirmed that GPX4 knockout mice exposed to CS had higher levels of free iron in bronchial epithelial cells and lung homogenates, airway and alveolar epithelial cell death, and inflammatory cells infiltration and pro-inflammatory mediator production in the broncho-alveolar lavage fluid (BALF), as compared with wild-type mice (92). In addition, GPX4 levels in the HBE of COPD patients were significantly lower than those of smokers and non-smokers, and GPX4 level was positively correlated with the ration of FEV_1_/FVC. Under electron microscopy, mitochondria in airway epithelial cells of COPD patients rather than nonsmokers exhibited ferrometastatic morphology characterized by aggregation and increased membrane density (88, 93).

Similarly, PM2.5, as an important exogenous risk factor for COPD, not only induces pathophysiological changes of COPD, but also aggravate inflammatory cell infiltration, inflammatory cytokine production, mucus secretion and goblet cell proliferation in CS-induced mouse COPD model (94). The main potential harm of PM2.5 is to induce COPD by producing a large amount of ROS to disrupt REDOX homeostasis (94). High levels of ROS and MDA, as well as depletion of GPX4, GSH and GSH-peroxidase were found in PM-induced mouse lung injury model and PM2.5-induced BEAS-2b cell damage. Hydrogen sulfide (H_2_S) treatment significantly reduced PM-induced emphysema and airway inflammation by inhibiting ferroptosis *in vivo* and *in vitro* through NRF2-PPAR pathway, while H_2_S treatment did not reverse lung injury and ferroptosis in NRF2 knockout mice (95, 96). These studies suggest that ferroptosis may play an important role in the development and progression of COPD. Ferroptosis induced by epithelial ferritin autophagy and free iron accumulation caused by smoking and environmental pollutants is a new pathogenesis of COPD. The administration of iron chelating agents, antioxidants and lipid peroxidation inhibitors will be expected to be used in the clinical management of COPD.

In summary, ferroptosis, which is associated with inflammation and cellular damage, initiates oxidative stress, which plays a major role in the detrimental impacts of PM2.5 on the respiratory system. Using ATAC-seq and RNA-seq techniques, researchers discovered that ferroptosis pathways accounted for the majority of the signaling pathways in PM2.5-induced mice lung damage. Subsequent investigations revealed that Fer-1 attenuated the observed decrease in cell viability, increased lipid peroxidation, and alterations in mitochondrial morphology after exposure to PM2.5 (97). Li et al. (98) identified that PM2.5 may induce pulmonary epithelial senescence and ferroptosis by modulating the USP3-SIRT3-P53 axis, thereby promoting the pathogenesis of pneumonia. NRF-2 gene plays an important role in lung damage caused by tiny particulate matter. Dong et al. (99) have demonstrated that the NRF-2 agonist tectoridin protects against PM2.5-induced lung injury by inhibiting ferroptosis-mediated lipid peroxidation. Several studies have also indicated that astaxanthin and melatonin can inhibit ferroptosis by activating the NRF-2 pathway, thereby alleviating PM2.5-induced lung injury (100, 101).

Similarly, other chemicals found in air pollutants can also cause lung inflammation. Sulfur dioxide in the air can induce oxidative stress and activate apoptotic pathways in rat lungs (102). Additional studies have shown that sulfur dioxide increases susceptibility to asthma and triggers acute exacerbations (103). Increased ozone levels in the air led to ROS accumulation and mitochondrial damage in the mice lungs, which in turn modified serum metabolites and causes or worsens respiratory inflammation (104, 105). Additionally, acute or chronic exposure to certain rare airborne pollutants (such as specific chemical gases and occupational environmental pollutants) can result in the onset of pulmonary environmental disorders. Liu et al. (106) demonstrated that beryllium sulfate (BeSO_4_) triggered iron-dependent lipid peroxidation, leading to lung injury. Furthermore, they found that the ferroptosis inhibitor DFO mitigated BeSO_4_-induced iron accumulation and lipid peroxidation in bronchial epithelial cells (106). Mao et al. (107) found that exposure to black carbon nanoparticles and cadmium triggered autophagy-related gene 5 (ATG5)-dependent ferroptosis, leading to the accumulation of ROS and iron ions in rat lungs, ultimately resulting in lung injury. Thus, there is a great medical and societal benefit to developing ferroptosis inhibitors for the prevention and treatment of air pollutant-induced lung injury.

### 3.2 Environmental pollution and ferroptosis in pulmonary fibrosis

Pulmonary fibrosis is a chronic progressive interstitial lung disease. Pulmonary fibrosis can be caused by several occupational variables such as metal dust, dust, radiation, viral infections, gastroesophageal reflux, or even unknown causes. The primary pathological characteristic of pulmonary fibrosis is the development of fibrotic foci, where fibroblasts transform into myofibroblasts, resulting in significant deposition of extracellular matrix, accumulation of collagen, deterioration of alveolar structure, and ultimately the destruction of normal lung structure (108). Studies have shown that a large amount of hemosiderin and iron-related oxygen free radicals accumulate in the alveolar macrophages and monocytes in IPF patients, and the expression of ferritin light chain in lung fibroblasts of IPF patients is significantly higher than that of healthy people (109). Excessive iron accumulation in the lung has been shown to associate with vascular abnormalities and pulmonary hypertension in the lungs of patients with IPF, which promotes ferroptosis in the pathophysiology of pulmonary fibrosis. Evidence suggests that ferroptosis in type II alveolar epithelial cells (ATII) is a potential pathological cause of IPF. In a mouse model of pulmonary fibrosis, the expression of GPX4 and FSP1 in lung tissues was reduced due to the administration of Bleomycin (BLM). Additionally, ATII cells in the lung tissues exhibited ferroptosis, which was characterized by mitochondrial shrinkage and an increased membrane density (110). BLM induces disruption of intracellular iron homeostasis through activation of the N-methyl-D-aspartate receptor, regulation of TFR1 and DMT1, and down-regulation of GPX4 and FSP1 levels, thus participating in the process of iron metabolism in PF (111). GPX4-deficient mice are more prone to lipid peroxidation and collagen deposition in the BLM-treated mice lungs. The administration of Trolox, a water-soluble form of vitamin E, can effectively slow down the progression of pulmonary fibrosis by decreasing BLM-induced lipid peroxidation in mice lungs (112). Study have shown that ambient air pollutants may contribute to the development of IPF through affecting the severity of disease, inducing acute exacerbations and hospitalization, and affecting mortality (113).

It was found that exposure to PM2.5 exacerbates BLM-induced pulmonary fibrosis in a time- and dose-dependent manner. This process is achieved through HO-1-mediated oxidative stress and NCOA4-mediated ferritin autophagy (114). Silicosis is a serious occupational disease. When patients are chronically exposed to industrial dust, the clearance and defense mechanisms of the respiratory system are disrupted, which in turn leads to fibrosis of lung tissue (115). Silica (SiO_2_) is the main cause of silicosis, and macrophages play a key role in the pathogenesis of silicosis. In silica-induced pulmonary fibrosis model, iron accumulation and GSH depletion trigger ferroptosis in macrophages and promote the secretion of pro-fibrotic cytokines to activate fibroblasts, thereby initiating fibrosis. SiO_2_-stimulated RAW264.7 cells resulted in an increase in intracellular lipid peroxidation levels, ROS and MDA expression, and a decrease in GPX-4 and System Xc- expression, which could be reduced by pretreatment with Fer-1 (116). Transforming growth factor β (TGF-β) is a potent fibrogenic factor that promotes extracellular matrix deposition by stimulating the growth of fibroblasts and inhibiting the degradation of matrix proteases, thus participating in the occurrence and development of IPF (117). HFL-1 cells treated with TGF-β showed a decrease in the size and cristae of mitochondria, increased expression of TFR, resulting in the accumulation of intracellular Fe^2+^, increased levels of ROS and MDA, decreased levels of GPX4, which are hallmarks of ferroptosis. Fer-1 can inhibit the increase of ROS, MDA, α-Smooth muscle actin (α-SMA), collagen I and the decrease of GPX4 induced by TGF-β, indicating that Fer-1 can inhibit ferroptosis and the differentiation of fibroblasts into myofibroblasts by reducing lipid peroxidation and promoting GPX4 expression (118, 119). After intratracheally instillation of SiO_2_, the expression of TGF-β, SLC7A11, TFR and other ferroptosis-related factors increased in mice lungs, while the expression of GPX4 was decreased. TGF-β inhibitor not only exerted an anti-fibrotic effect, but also reduced the level of ferroptosis (120). Therefore, blocking TGF-β signaling pathway by anti-oxidant and iron chelator could become a potential therapeutic strategy in reducing pulmonary fibrosis and prolonging the survival of PF patients (121). Hydroxyproline (HYP), a unique amino acid in collagen, is a non-essential amino acid and one of the major components of collagenous tissue (122). In a study of radiation-induced pulmonary fibrosis in mice, the experimental group showed a significant increase in serum inflammatory cytokine levels, as well as a significant increase in HYP and collagen deposition in the lung tissues compared to the control group. In addition, the lung tissue sections exhibited obvious fibrotic features, with a marked increase in ROS and a decrease in GPX4 in mouse lung tissue. Lip-1, an iron mutation inhibitor, inhibited collagen deposition, HYP and ROS accumulation, serum inflammatory markers release, and GPX4 elevation in fibrotic mice lung tissues (123). Therefore, ferroptosis plays an important role in the occurrence and development of pulmonary fibrosis, providing a new idea for the treatment of pulmonary fibrosis.

### 3.3 Environmental pollution-induced ferroptosis in lung cancer

Lung cancer, a leading cause of cancer mortality globally, is exacerbated by oxidative stress caused by ROS and nitrogen species (RNS) from air pollutants, by increasing lung inflammation and carcinogenesis (72). Prolonged exposure to air pollution, particularly fine particulate matter, has been linked to an increased risk of lung cancer, particularly in areas where air pollution is severe (124, 125). Notably, indoor pollution has been connected to a higher incidence of lung cancer in non-smoking women (126). Recurrence and drug resistance continue to be major problems despite a variety of treatments, including surgical, radiological, chemotherapeutic, immunotherapeutic, and genetic therapy (109). Nevertheless, majority of patients experience drug resistance and recurrence during the treatment, leading to deterioration of the disease. Hence, it is crucial to investigate the underlying mechanism of the development, progression, and drug resistance in lung cancer.

Research reveals that disrupted iron metabolism and inhibition of ferroptosis are central to lung cancer progression. Elevated ferritin levels have been found in serum, BALF and breath condensate of individuals with lung cancer (127). Furthermore, lung cancer cells may prevent ferritin buildup through a variety of mechanisms and are resistant to oxidative damage brought on by the high oxygen levels in the lungs (72). Elevated ferritin in biological fluids of lung cancer patients suggests iron dysregulation, and mechanisms to resist oxidative stress and avoid ferritin accumulation are prominent in non-small cell lung cancer (NSCLC) cells (128, 129). Furthermore, a study reported that 88% of NSCLC patients exhibited elevated TFR1 levels, leading to increased intracellular iron accumulation. Notably, iron homeostasis was maintained at levels conducive to tumor survival, thereby preventing ferroptosis induced by iron overload. This phenomenon was primarily attributed to EGFR activation, which allowed cancer cells to acquire iron while evading ferroptotic cell death (130). For instance, NSCLC cells inhibit ferroptosis through upregulating SLC7A11, driven by SOX2 transcription factor, conferring resistance to oxidative damage. Iron homeostasis and resistance to ferroptosis are further complicated by the high expression of TFR1 and GPX4 in these cells, although proteins such as FSP1 encourage cellular proliferation by preventing ferritin deposition even in the absence of GPX4 activity (131). Interestingly, the mechanisms underlying ferroptosis resistance are not solely intrinsic; they are also influenced by external environmental factors. For example, exposure to environmental pollutants, such as PM2.5 (132), can exacerbate oxidative stress and disrupt iron metabolism, further complicating the regulation of ferroptosis in lung cancer. Studies conducted in regions with a high prevalence of lung cancer have linked bituminous coal pollution to specific genetic alterations, such as haptoglobin depletion and thioredoxin 2 (TXN2) overexpression, which contribute to increased treatment resistance by modifying ferroptotic pathways (133). A toxicogenomic-based study examined the effects of hydrocarbon mixtures, including polycyclic aromatic hydrocarbons (PAHs), on the molecular mechanisms of lung cancer. This study found that, in the early stages of cancer, pollutants induce oxidative stress, potentially leading to the upregulation of ferroptosis. However, as the cancer progresses, cells adapt by modulating iron metabolism and antioxidant defenses, such as upregulating GPX4 and SLC7A11, to suppress ferroptosis and enhance survival (134). In addition, a recent study demonstrated that exogenous CO can regulate ROS and GPX4 levels to promote ferroptosis in NSCLC, suggesting a novel therapeutic strategy for cancer treatment (135).

The intricate balance between iron metabolism and ferroptosis resistance in lung cancer, shaped by both intrinsic cellular mechanisms and external environmental factors, underscores the potential for therapeutic interventions targeting these pathways. Environmental pollutants, by modulating ROS levels and disrupting iron homeostasis, can either drive cells toward ferroptosis or exacerbate tumor progression, depending on the specific context. This duality highlights the critical importance of harnessing ferroptosis in cancer treatment strategies. One promising treatment strategy is to target the regulators of ferroptosis. By increasing susceptibility to oxidative stress and iron buildup, ferroptosis inducers like Erastin, RSL3, and FSP1 inhibitors have the potential to overcome resistance in non-small cell lung cancer. As proven in NSCLC xenograft models, combination treatments with ferroptosis inducers and conventional medications (such as cisplatin and sorafenib) had synergistic anti-tumor effects (51). For example, erastin enhances intracellular ROS formation and inhibits NSCLC cell proliferation via p53 activation and SLC7A11 suppression (136). The underlying mechanism of conventional anticancer drugs has been gradually found to be intimately linked to ferroptosis. In NSCLC cells that are resistant to cisplatin-based chemotherapy regimens, ferroptosis can be efficiently induced by the combination of Erastin and the traditional anticancer medication sorafenib. In a xenograft tumor model of naked mice, erastin and sorafenib successfully inhibited the growth of drug-resistant NSCLC cells. The lowering of System Xc? expression, a downstream target of NRF2/xCT, is probably the cause of this inhibition (137). A strategic supplement to traditional treatments for lung cancer, inhibition of these pathways by certain ferroptosis-inducing drugs may slow the growth and spread of drug-resistant cancer cells.

## 4 Role of ferroptosis in specific environment-induced lung injury

The health of populations not only depends on the availability of clean air, water, food, and sanitation (138, 139), but also the stability of the environment. In recent years, Individuals who need to work in a specific environment or have sudden exposure can causes specific acute and chronic physiological responses in humans (140). The lung is the primary interface to exchange substances with the environment, so drastic changes in the environment can directly or indirectly damage the lung tissue (Table 2).

**TABLE 2:** Specific environment-induced ferroptosis in lung diseases.

Specific environment	Models of diseases	Mechanism	References
MPs	Lung injury	Ferritin autophagy	(162, 163)
PS-MP	Aggravation of eosinophilic allergic asthma	NF-κB mediated ferroptosis	(164)
Nanoplastics	Lung injury	ROS-dependent endoplasmic reticulum stress	(163)
PS-MP	Pulmonary fibrosis	cGAS/STING signaling	(165)
High-altitude and low-pressure	Pulmonary edema	NRF-2 mediated ferroptosis	(178)
Smoke	Pulmonary injury	Glutathione metabolism mediated ferroptosis	(187)
		Lipid peroxidation	(181, 188, 189)
		JNK signaling pathway	(190)
Seawater	Pulmonary epithelial cells injury	HO-1 mediated oxidative stress	(197)
		Nrf-2 mediated ferroptosis	(198, 199)

### 4.1 MP

During the second United Nations Environment Assembly in 2015, plastic pollution was highlighted as a significant concern in environmental and ecological sciences (141). Microplastics (MPs), defined as plastic particles smaller than 5 mm, include materials such as polyethylene, polypropylene, polystyrene, and polyester (142). These particles are ubiquitous in aquatic, atmospheric, and terrestrial ecosystems and possess a high surface area and hydrophobicity (143), allowing them to absorb environmental pollutants and act as vectors for pathogenic microorganisms, thereby posing risks to human health (144). Research indicates that MPs can accumulate in human respiratory systems, with studies showing high concentrations of MPs in the sputum of individuals with respiratory disorders (145–147). This accumulation may contribute to the pathogenesis of lung diseases such as asthma and chronic obstructive pulmonary disease (COPD) by disrupting alveolar surfactants and inflammatory responses (148–151).

Experimental findings reveal that exposure to MPs can induce oxidative stress and ferroptosis, a form of regulated cell death characterized by lipid peroxidation (152, 153). For instance, studies on carp demonstrated that MPs activate the NF-κB signaling pathway, leading to ferroptosis and intestinal damage (154). In aged mice, MPs were shown to exacerbate cognitive dysfunction through similar mechanisms (155). In rat models, exposure to polystyrene MPs increased levels of TGF-β, TNF-α, and NF-κB in lung tissues in a dose-dependent manner (156–158). Intratracheal administration of nanoplastics (NPs) activated the NF-κB/NLRP3/caspase-1 pathway, elevating oxidative stress and inflammation, thereby inducing lung injury (159). Antioxidants such as N-acetylcysteine have demonstrated protective effects against NP-induced lung damage. Recent studies have also linked MPs to alterations in mitochondrial function, with findings showing decreased cell viability and increased mitochondrial reactive oxygen species (mtROS) production in lung epithelial cells treated with NPs (160). Additionally, polystyrene was found to enhance the conversion between ascorbic acid and deoxy-ascorbic acid, raising hydroxyl radical levels in simulated lung fluid and compromising the antioxidant capacity of bronchial epithelial cells (161). Yang et al. discovered that MPs promoted lung epithelial cell damage through ferritin autophagy mediated by oxidative stress-driven mitochondrial damage, and this injury could be alleviated by the ferroptosis inhibitors ferrostatin-1 and deferoxamine (162, 163). Wei et al. (164) discovered that the use of metabolomics technologies revealed that exposure to (PS-MP) aggravates eosinophilic allergic asthma in mice through various ferroptosis pathways, including the NF-κB pathway. Emerging evidence suggests that MPs accelerate lung disease progression by disrupting redox homeostasis and promoting ferroptosis through various signaling pathways, including the cGAS/STING pathway (165). Overall, microplastics represent a critical threat to pulmonary health, facilitating the development of respiratory diseases through their complex interactions with cellular and molecular pathways.

### 4.2 High altitude

The special environment of low pressure, hypoxia, and low temperature in plateau region cause a reduction in the partial pressure of oxygen in the blood, leading to tissue and organ hypoxia. High altitude pulmonary edema (HAPE) is a form of non-cardiogenic pulmonary edema that happens in individuals who are not acclimated to high elevations exceeding 2,500 meters (166). HAPE exhibits a fast and escalating start, with a fatality rate ranging from 36 to 90%. The common clinical manifestations are dyspnea, cough, cyanosis, exercise intolerance, and pink foam sputum (167). Recent research indicates that the pathogenesis of HAPE is associated with hypoxic pulmonary hypertension, oxidative stress, increased pulmonary vascular permeability, inflammatory response, impaired alveolar fluid clearance, and genetics factors (168). Particularly, NO synthesis is reduced in hypoxic conditions, leading to a diminished inhibitory effect on sympathetic nerve. More precisely, reduced NO synthesis under hypoxic conditions weakened the inhibitory effect on the sympathetic nervous system, leading to pulmonary vasoconstriction and subsequent pulmonary hypertension (169–171). Additionally, hypoxia stimulated NF-κB gene transcription, resulting in the production of pro-inflammatory cytokines (172). Hypoxia also increased the production of ROS, thereby enhancing oxidative stress (173), which is closely linked to the inflammatory process. Studies under hypoxic conditions have shown that ERK-1/2 activation, which subsequently activated NF-κB, induced oxidative stress via NOX4-produced H_2_O_2_ (174). There is a positive feedback loop between oxidative stress and inflammation, exacerbating acute lung injury (174, 175). Nevertheless, the precise mechanism by which the disease develops is not yet fully understood, and the prevention and treatment strategies are also lack pertinence. Although the plateau stepwise approach is the most effective means of preventing and treating HAPE, it requires a longer period of time and the short-term exposure is difficult to achieve preventive effects. Therefore, its application in emergency disaster relief and emergency military operations is limited.

In a model of high-altitude and low-pressure-induced pulmonary edema in rats, acute low-pressure and low-pressure exposures for 48 h were found to cause severe lung damage with edema, alveolar hemorrhage, TNF-α and IL-1β production, and inflammatory cell infiltration (176, 177). Disruption of mitochondrial cristae, reduction of mitochondrial volume, and increase in mitochondrial electron density and membrane density in alveolar epithelial cells were observed by electron microscopy. In addition, elevated ROS, MDA and depletion of SLC7A11, GPX4 and HO-1 were detected in rat lung tissues. Following treatment of drugs with antioxidant effects, lung injury was ameliorated by inhibiting oxidative stress and resisting ferroptosis through activation of NRF2 (178). A comprehensive study utilizing several omics approaches, including genomic and proteomic analyses, investigated the differences in ferroptosis signaling and iron homeostasis among ascending lowlanders, acclimatized lowlanders, and indigenous high-altitude populations (179, 180). Hence, drugs inhibiting ferroptosis can be validated and utilized as an effective therapeutic strategy for the clinical management of HAPE.

### 4.3 Smoke inhalation injury

Smoke inhalation injury (SII) is a chemical injury of the trachea and lung parenchyma caused by the inhalation of hot air, steam, smoke particles, volatile chemicals and toxic gases. It is a major cause of morbidity and mortality in fire victims and one of the risk factors for injuries to rescuers (181). SII can trigger an inflammatory response, leading to an increased requirement for fluid resuscitation and a higher likelihood of developing pulmonary problems such as pulmonary atelectasis, pneumonia, and ARDS. Although the pathogenesis of SII has not yet been fully elucidated, it has been shown that an imbalance between oxidative stress and antioxidant defense, as well as an inflammatory cascade response, are involved (182). The predominant pathological process is the activation of the NF-κB pathway by smoke particles (183, 184), leading to the expression of pro-inflammatory cytokines. These cytokines, in turn, activate NF-κB, creating a vicious cycle that exacerbates lung injury and produces reactive oxygen species ROS and NO (185). NO molecules bind to cellular superoxide, creating a potent oxidant that can damage cells and DNA (183). The survival rate of burn patients has greatly improved in recent years due to advancements in fluid resuscitation, surgical wound treatment, wound dressings, antibiotic applications, and nutritional support (186). However, inhalation injury significantly increases burn complications and mortality, and there is evidence that burn patients with SII will experience a nearly 24-fold increase in mortality (184). The clinical management of SII remains predominantly supportive and targeted therapy remains limited.

Using microarray technology, researchers quantified the differentially expressed lncRNAs and mRNAs in the lungs of SII mice and normal control mice, and performed GO and KEGG pathway analyses. GO analysis showed that the differential genes had oxidoreductase activity and were involved in redox reactions, and KEGG analysis showed that the differential genes were involved in glutathione metabolism and ferroptosis (187). Nevertheless, there is a lack of definitive evidence suggesting that ferroptosis plays a role in the development of smoke inhalation injury. Several investigations have shown that smoke inhalation injury is associated with the accumulation of lipid peroxides, an imbalance in oxidative stress, and the excessive accumulation of iron ions. In cotton smoke inhalation-induced rat lung injury model, membrane lipid peroxidation, increased MDA expression, and decreased GSH expression were found in lung tissues at both 6 and 24 h post-injury, suggesting that ferroptosis may occur during SII (181). Similarly, in a sheep model of smoke inhalation injury, the application of the iron chelating agent DFO was discovered to reduce smoke-induced airway damage and mitigate the systemic inflammatory response (188, 189). In a mouse model of lung injury caused by inhaling cotton smoke, the researchers found that damage to the airway epithelium and increased production of mucus in mice were connected to the activation of the JNK signaling pathway (190), which is closely associated with ferroptosis (191). Therefore, targeting ferroptosis may provide new insights into the diagnosis and treatment of SII.

### 4.4 Seawater inhalation

More than 400,000 people die from drowning annually worldwide, with deaths from seawater drowning ranking third in accidental deaths (192). Seawater inhalation can rapidly lead to a severe pulmonary inflammatory response, resulting in seawater inhalation lung injury (SW-ALI), which is characterized by critical condition, rapid progression, and high mortality (193). Seawater aspiration, like other stress conditions such as trauma, burns, and sepsis, can induce ALI/ARDS, with hypoxemia being the primary pathophysiological alteration. Despite advances in organ-protective therapeutic techniques, clinical treatment of SW-ALI still lacks targeted and effective measures, and the mortality rate remains high. In a mouse model of seawater drowning, researchers observed activation of the NF-κB pathway, leading to significant infiltration of inflammatory factors such as TNF-α and inflammatory cells into lung tissue (194). Additionally, in a rat model of acute lung injury induced by seawater instillation, the accumulation of ROS in alveolar epithelial cells and activation of endoplasmic reticulum stress has been found, ultimately resulted in apoptosis of alveolar epithelial cells (195). Furthermore, activation of the JNK signaling pathway in mitochondria was implicated in autophagy and oxidative stress in seawater inhalation-induced acute lung injury (196).

In the cellular model, there was an upregulation of ROS expression and concurrent impairment of mitochondria. In subsequent experiments, HO-1 was upregulated, exerting anti-inflammatory and antioxidant effects that were protective (197), which provided sufficient conditions for the occurrence of ferroptosis. It was found that seawater exposure altered the morphology of mouse lung epithelial cells MLE-12 and reduced cell viability in a time-dependent manner. Intracellular ROS and MDA were significantly increased, while GSH levels and SOD activity were significantly decreased. Fer-1, an inhibitor of ferroptosis, significantly ameliorated the decrease in cell viability and reversed the impairment of cellular GSH and SOD. Subsequent studies found that activation of NRF2 inhibited the PTGS2, a ferroptosis-activating gene, to protect against SW-ALI, inhibiting ferroptosis and repairing mitochondrial function (198). Conversely, in mice lacking the NRF2 gene, seawater drowning increases MDA levels and decreases GSH levels in mice lung tissue, aggravating lung injury. In NRF2 gene deletion cells, increased intracellular ROS and ferroptosis levels were observed, ultimately resulting in increased cell death (199).

## 5 Conclusions and outlook

Ferroptosis is closely related to dysregulation of cellular redox homeostasis, and most respiratory diseases are associated with pulmonary oxidative stress. Similarly, drastic environmental changes can also induce oxidative stress-mediated ferroptosis in the lungs. Therefore, it may be valuable to study the role of ferroptosis in lung injury. At present, there are numerous challenges in the management of respiratory illnesses using conventional medications, including long-term hormone therapy for asthma and COPD, adverse effects of radiotherapy for lung cancer, and the poor therapeutic effect of anti-inflammation and anti-fibrosis on IPF. Current studies suggest that ferroptosis is crucial for lung diseases and the application of ferroptosis inhibitors may attenuate the damaged lungs or ameliorates tumorigenesis. Ultimately, further investigation should prioritize the discovery of exceptionally targeted ferroptosis inducers or inhibitors, with the aim of translating them into clinical practice. The combination of conventional drugs with ferroptosis inducers or inhibitors for the treatment of respiratory diseases may compensate for the drawbacks of conventional drugs and bring benefits to patients. Moreover, ferroptosis represents not only a critical mechanism in pulmonary diseases but also a distinct form of cell death, setting it apart from other cell death pathways. This uniqueness positions ferroptosis as a promising frontier for future research and therapeutic innovation.
